# Preserved *Ex Vivo* Inflammatory Status in Decidual Cells from Women with Preterm Labor and Subclinical Intrauterine Infection

**DOI:** 10.1371/journal.pone.0043605

**Published:** 2012-08-22

**Authors:** Violeta Castro-Leyva, Aurora Espejel-Nuñez, Gerardo Barroso, Veronica Zaga-Clavellina, Arturo Flores-Pliego, Iyari Morales-Mendez, Silvia Giono-Cerezo, Scott W. Walsh, Guadalupe Estrada-Gutierrez

**Affiliations:** 1 Department of Infectology, Instituto Nacional de Perinatologia Isidro Espinosa de los Reyes, Mexico City, Mexico; 2 Posgrado en Ciencias Quimico-Biologicas, Escuela Nacional de Ciencias Biologicas, Instituto Politecnico Nacional, Mexico City, Mexico; 3 Department of Biochemistry and Molecular Biology, Instituto Nacional de Perinatologia Isidro Espinosa de los Reyes, Mexico City, Mexico; 4 Clinical Research Division, Instituto Nacional de Perinatologia Isidro Espinosa de los Reyes, Mexico City, Mexico; 5 Department of Cellular Biology, Instituto Nacional de Perinatologia Isidro Espinosa de los Reyes, Mexico City, Mexico; 6 Department of Obstetrics and Gynecology and Physiology and Biophysics, Virginia Commonwealth University Medical Center, Richmond, Virginia, United States of America; Columbia University, United States of America

## Abstract

**Objective:**

To compare the inflammatory response preserved *ex vivo* by decidual cells isolated from women who experienced preterm labor with and without subclinical intrauterine infection.

**Methods:**

Fetal membranes were obtained after cesarean section from 35 women who delivered before 37 weeks of gestation following spontaneous preterm labor, with no clinical evidence of intrauterine infection. Decidua was microbiologically tested and cultured. Concentrations of anti-inflammatory cytokines (IL-2, IL-4, IL-10), pro-inflammatory cytokines (IL-6, IL-8, IL-1β and TNF-α), and matrix metalloproteinases (MMP-1, MMP-2, MMP-3, MMP-7, MMP-8, MMP-9) were measured in the supernatants using Bio-Plex, and prostaglandin E_2_ (PGE_2_) was measured by enzyme immunoassay.

**Results:**

Subclinical infection was confirmed in 10 women (28.5%). Microorganisms isolated were *Ureaplasma urealyticum* (4), group B streptococci (3), *Gardnerella vaginalis* (1), and *Escherichia coli* (2). We found a significant increase of pro-inflammatory cytokines and a significant decrease of anti-inflammatory cytokines in supernatants from decidual cells obtained from women with preterm labor and subclinical intrauterine infection compared to women without infection. Secretion of MMP-1, MMP-8, MMP-9 and PGE_2_ was significantly higher in infected women. Secretion of IL-8 by decidual cells from infected women persisted upon repeated *in vitro* culture passages.

**Conclusions:**

Almost 30% of idiopathic preterm labor cases were associated with subclinical intrauterine infection, and decidual cells isolated from these cases preserved an *ex vivo* inflammatory status after *in vivo* bacterial exposure.

## Introduction

Preterm birth is an important perinatal health problem worldwide. The number of preterm births is approximately 12.9 million per year representing 9.6% of births [1]. Given that 1 out of every 10 births is premature, the Institute of Medicine of the National Academies recommends a multidisciplinary research agenda aimed at improving the prediction and prevention of preterm labor (PTL) and assuring healthy outcomes [2].

Intrauterine bacterial infections are considered an important cause of preterm birth [3], and may cause devastating neonatal consequences, such as cerebral palsy [4]. Bacteria proliferate in the lower genital tract and may ascend to the intrauterine cavity [5], where they can trigger an inflammatory response in decidual cells, resulting in secretion of pro-inflammatory cytokines, matrix metalloproteinases (MMPs) and prostaglandins [6], [7]. Most of these types of infections are subclinical in nature and cannot be detected without amniotic fluid analysis [5], [8]. Evidence to support a role of subclinical intrauterine infection during preterm birth includes the presence of histological chorioamnionitis, clinical infection after preterm birth, positive amniotic fluid cultures, association of lower genital tract microorganisms with preterm birth, and biochemical markers of infection [9].

Although it is well known that inflammatory mediators are increased during PTL, and further increased in cases with intrauterine infection [10], [11], decidual secretion of these molecules during subclinical intrauterine infection has not been studied. Inflammatory response triggered in decidual cells is important because they are the conduit through which microorganisms gain access to the intrauterine cavity. They first infect the decidua, and then move to the chorion, amnion, amniotic cavity, and eventually the fetus [3]. The following study was conducted to compare the inflammatory response of cultured decidual cells obtained from women with PTL with and without subclinical intrauterine infection to determine if the inflammatory response associated with subclinical infection was preserved *ex vivo*.

## Results

### Incidence of Subclinical Intrauterine Infection and Causal Microorganisms

**Figure 1:**
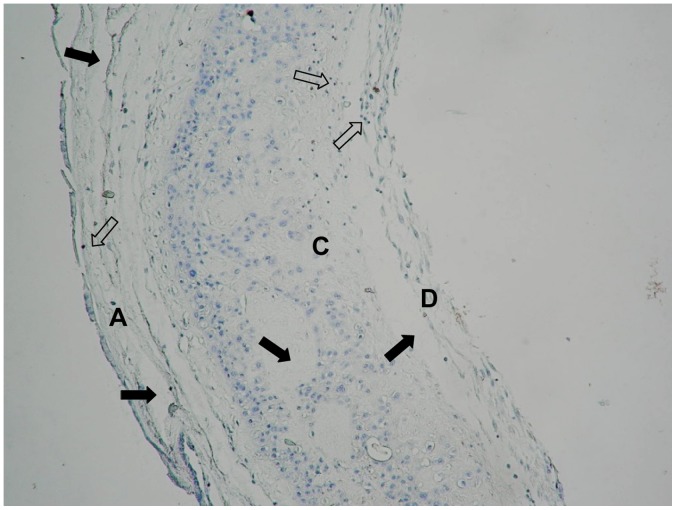
Neutrophil infiltration and extracellular matrix damage in amniochorion from a woman with subclinical intrauterine infection. Representative immunohistochemistry of the fetal membranes from a woman with preterm labor and subclinical intrauterine infection. Open arrows show neutrophil infiltration, and filled arrows indicate damage to the extracellular matrix arrangement in amnion (A), chorion (C), and decidua (D). Light microscopy, hematoxilin stain.

Ten of the 35 women enrolled in the study (28.5%) had positive cultures for pathogens, confirming the presence of subclinical intrauterine infection. Microorganisms identified included: *Ureaplasma urealyticum* (4 cases), group B streptococci (3 cases), *Escherichia coli* (2 cases), and *Gardnerella vaginalis* (1 case). Local tissue inflammation was confirmed by the presence of neutrophil infiltration in amnion, chorion and decidual layers in all infected cases (Figure 1). In contrast, neutrophil infiltration was observed only in one case with negative culture. Demographic and clinical data of the patients are listed in Table 1. There were no statistically significant differences between groups for maternal weight, gestational age at delivery or newborn weight (Table 1).

**Table 1:** Demographic and clinical characteristics of the women with preterm labor included in the study.

	Without infection (n = 25)	With infection (n = 10)	*P* value
**Maternal age (y)**	29.04±1.0	28.30±2.1	0.72
**Gestational age at delivery (wk)**	33.0±1.9	32.9±2.5	0.27
**Newborn weight (g)**	2202±267	2346±295	0.29

### Pro- and Anti-inflammatory Cytokine Profiles

**Figure 2:**
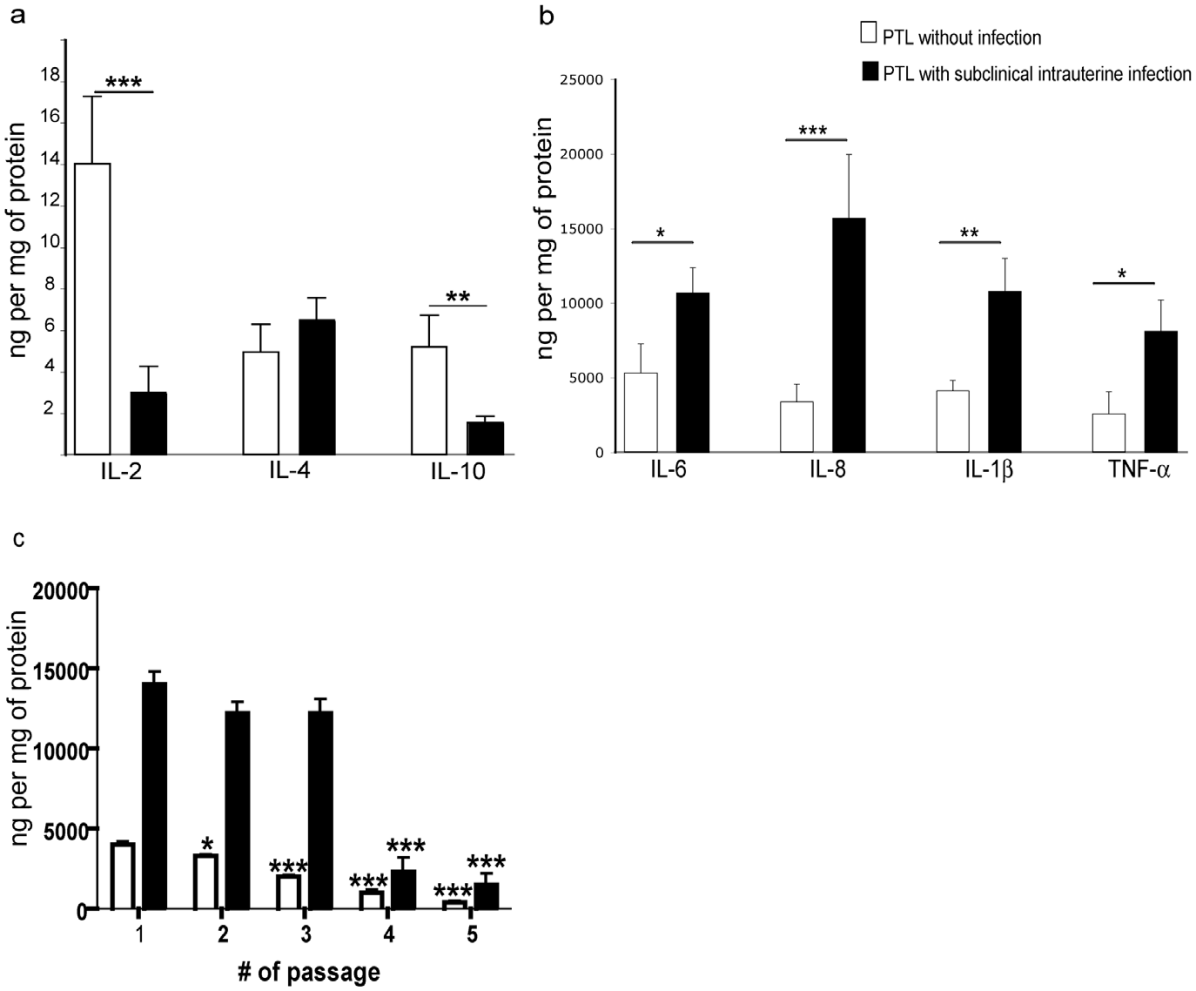
Profiles of cytokines secreted by cultured decidual cells from women with and without subclinical intrauterine infection. Concentrations of: **a)** anti-inflammatory cytokines, **b)** pro-inflammatory cytokines, and **c)** IL-8 in the supernatants of decidual cell cultures from women with preterm labor (PTL), with (n = 10) or without subclinical intrauterine infection (n = 25). Data in **c)** represent concentrations of IL-8 in the supernatants of progressive culture passages. Data are presented as mean ± SD; experiments were performed in duplicate. ***P<0.001, **P<0.005, *P<0.05 as compared to passage 1 for each group.

The anti-inflammatory cytokine quantification revealed a significant decrease of IL-2 and IL-10 in the supernatants of decidual cells from women with subclinical intrauterine infection (*P*<0.001 and *P*<0.005, respectively) compared to non-infected women, whereas IL-4 was not significantly altered (Figure 2a). In contrast, cytokines that promote a pro-inflammatory microenvironment were significantly increased in supernatants from decidual cells of women with intrauterine infection. lL-8 and TNF-α were increased 3-fold compared to non-infected women (*P*<0.001 and *P*<0.05, respectively) and IL-1β and IL-6 were increased 2-fold (*P*<0.005 and *P*<0.05, respectively) (Figure 2b).

### Persistence of IL-8, an Inflammatory Mediator, in Progressive Decidual Cell Passages

Secretion of IL-8 from decidual cells in culture was significantly greater for cells obtained from women with PTL and subclinical infection than women with PTL and no infection. The increased secretion of IL-8 persisted during repeated passages and did not significantly decline until the 4^th^ passage. In contrast, secretion of IL-8 from cells of women without infection was significantly less by the 2^nd^ passage (Figure 2c).

### Matrix Metalloproteinase and Prostaglandin Secretion Profiles

**Figure 3:**
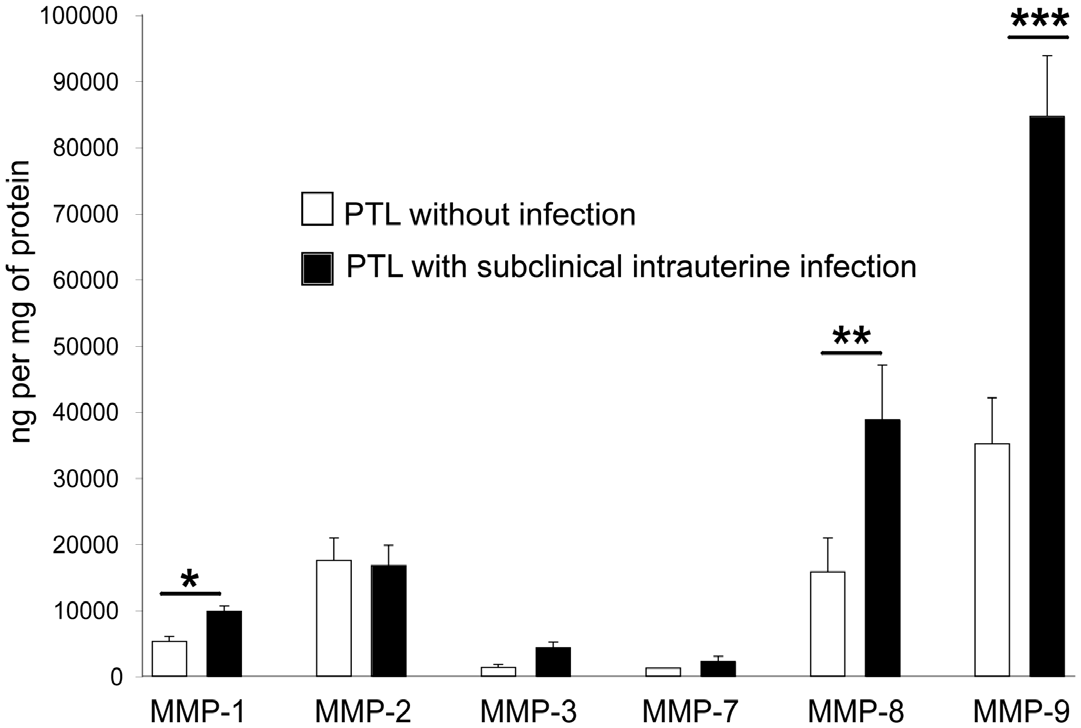
Profiles of matrix metalloproteinases secreted by cultured decidual cells from women with and without subclinical intrauterine infection. Concentrations of matrix metalloproteinases in the supernatants of decidual cells from women with preterm labor (PTL), with (n = 10) or without subclinical intrauterine infection (n = 25). Data are presented as mean±SD; experiments were performed in duplicate. ***P<0.001, **P<0.005, *P<0.05 as compared to PTL without infection.

**Figure 4:**
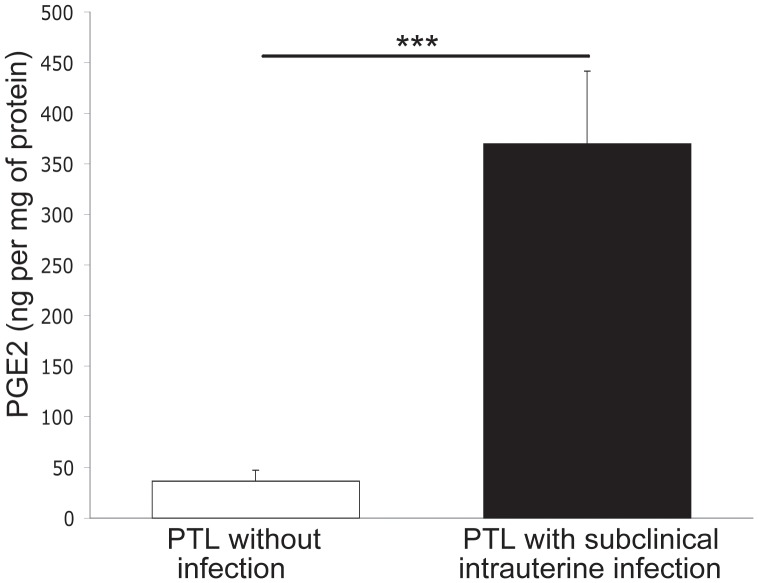
Prostaglandin E_2_ secreted by cultured decidual cells from women with and without subclinical intrauterine infection. Concentrations of prostaglandin E_2_ in the supernatants of decidual cell cultures from women with preterm labor (PTL), with (n = 10) or without subclinical intrauterine infection (n = 25). Data are presented as mean±SD; experiments were performed in duplicate. ***P<0.001.

Evaluation of MMP secretion profiles demonstrated that MMP-1, MMP-8, and MMP-9 were significantly increased in supernatants of decidual cells isolated from women with subclinical intrauterine infection, compared to cells from non-infected women (*P*<0.05, *P*<0.005, and *P*<0.001, respectively) (Figure 3). Concentrations of PGE_2_ were also significantly increased in cases with subclinical intrauterine infection demonstrating a 10-fold increase over cases without infection (*P*<0.001) (Figure 4).

## Discussion

Clinically overt intrauterine infection is an important cause of PTL and is characterized by increased production of inflammatory mediators by gestational tissues [7], [12]. Subclinical intrauterine infection also occurs and a large body of evidence suggests it causes preterm birth [5], [8], however, inflammation associated with subclinical infection is difficult to detect during pregnancy. In this study, we compared the inflammatory response elicited by primary cultures of decidual cells isolated from cases with PTL and subclinical intrauterine infection to cases with PTL and no infection to determine if they could be differentiated by their inflammatory response.

Our results demonstrated that almost 30% of pregnant women with PTL who showed no signs of clinical infection have subclinical intrauterine infection. *Ex vivo* cultured decidual cells from these women secreted increased amounts of pro-inflammatory cytokines (IL-6, IL-8, IL-1β, and TNF-α). Increased secretion of these inflammatory cytokines was most likely a consequence of the previous *in vivo* bacterial contact. In contrast, the anti-inflammatory response as assessed by IL-2 and IL-10 secretion was decreased. These results demonstrate a pro-inflammatory microenvironment exists in women with subclinical intrauterine infection and PTL.

An increase of pro-inflammatory cytokines in gestational tissues has been linked to increased production of matrix metalloproteinases [13], [14], [15] and prostaglandins [16], [17]. We found increased secretion of MMP-1, MMP-8, and MMP-9 by decidual cells in women with subclinical intrauterine infection. Such an increase in MMP’s may result in premature cervical dilatation leading to PTL. This notion is supported by previous work showing that matrix metalloproteinases exert a biological impact on cervical dilatation [18], [19], [20]. We and other investigators have demonstrated that these enzymes are directly implicated in damage to the extracellular matrix that provides tensile strength of the fetal membranes [6], [21], [22], [23], [24]. In this study, we observed structural damage to the connective tissue in amnion, chorion and decidua. This could result in rupture of the amnion and chorion leading to PTL. The finding that decidual cells isolated from women with subclinical intrauterine infection secrete high levels of PGE_2_, suggests that this prostaglandin is implicated in the uterine contractions that initiate PTL. Inflammatory mediators secreted by decidual cells during subclinical infection could participate in an autocrine and paracrine signaling network to amplify the inflammatory response in the intrauterine microenvironment triggering PTL.

Although an inflammatory signaling network has been suggested before [7], to our knowledge this is the first study that shows secretion of inflammatory mediators by cells directly isolated from infected women. In addition, our work demonstrates that decidual cells preserve their inflammatory status for at least 3 weeks after isolation. This suggests that in utero bacterial infection may program these cells to produce increased amounts of inflammatory mediators. These findings could explain controversial data concerning the efficacy of antimicrobial therapy to reduce the rate of preterm birth [3], [25].

The use of molecular-based techniques like bacterial 16S rDNA PCR to accurately estimate the prevalence of subclinical intra-amniotic infection has been proposed, but requires sequencing of the PCR products to identify specific bacteria, which is not feasible in the clinical setting. In addition, results from these studies are controversial because while some find that molecular techniques improve detection of bacteria [26], [27], others reveal that microbial prevalence is higher only when PCR is combined with culture experiments [28]. It is important to note that all of these studies used amniotic fluid, so they evaluated bacterial invasion of the amniotic cavity. As a consequence, the presence of the bacterial “footprints” detected by PCR was usually associated with adverse outcome [5].

When fetal membranes from elective cesarean section at term were tested, up to 70% of them were positive for bacterial 16S rDNA [29], which may indicate a low-level of colonization that did not elicit an inflammatory response resulting in preterm birth [5]. Although our study has the limitation that it was conducted using only culture to detect infection, the high prevalence of subclinical intrauterine infection that was found, and the fact that bacterial isolation was well correlated with the presence of inflammatory infiltrate in the fetal membranes and elevated secretion of inflammatory markers by decidual cells, validate that the subclinical infection rate was not underestimated. Although we evaluated a wide selection of bacterial species, the presence of previously unrecognized, uncultivated or difficult-to-cultivate species [30] cannot be ruled out. This may explain the presence of inflammatory infiltrate in the one fetal membrane that was negative by culture.

Ongoing work in our laboratory is focusing on: 1) the differences among bacterial strains implicated in clinical and subclinical intrauterine infection, 2) the effect of antibiotics on inflammatory mediator secretion by decidual cells in culture, and 3) the potential role of epigenetic regulation, and specifically DNA methylation, on inflammatory gene expression in these cells.

## Materials and Methods

### Ethics Statement

This study was approved by the Institutional Review Board of the Instituto Nacional de Perinatologia Isidro Espinosa de los Reyes in Mexico City (Register 212250-22711). The mothers signed the informed consent.

### Specimens

Fetal membranes were collected from 35 women who delivered by cesarean section following spontaneous PTL with intact membranes, and no clinical evidence of intrauterine infection. Preterm labor was defined as regular uterine contractions in combination with a cervical dilatation of ≥2 cm prior to completion of 37 weeks of gestation. Women with premature rupture of the membranes, preeclampsia, obesity, twin pregnancies or who were smokers were excluded from the study.

Fetal membranes were obtained under aseptic conditions and transported to the laboratory within 10 min of delivery to be microbiologically tested for subclinical intrauterine infection. Decidual swabs were rolled onto Columbia agar with 5% sheep blood, which was used as a primary isolation medium for fastidious and non-fastidious aerobic microorganisms. Appropriate selective media for detection of specific pathogens, e.g., MacConkey II agar (*E. coli*), Gardnerella selective agar with 5% human blood (*G. vaginalis*), potato dextrose agar (*C. albicans*), agar with 5% human blood (group B streptococci), and Chocolate II agar (*N. gonorrhoeae*), were used. A CDC anaerobe 5% sheep blood agar plate was streaked to isolate obligate and facultative anaerobes as well as microaerophilic bacteria, using a GasPak EZ anaerobic system. All culture media were purchased from BD (Germany) and were incubated following manufacturer’s instructions. An additional swab was inoculated into Urea-Arginine LYO 2 broth (bioMérieux, Switzerland) to detect decidual infection due to mycoplasma species. A portion of each specimen was formalin-fixed and paraffin-embedded to evaluate underlying inflammation by immunohistochemistry.

### Isolation and Culture of Decidual Cells

The decidua was scraped from the chorion under aseptic conditions, and decidual cells were isolated as previously described [31]. Leukocytes were eliminated by magnetic cell sorting using a CD45 magnetically labeled monoclonal antibody, LS columns and a VarioMACS magnet (Miltenyi Biotec, Germany) according to the manufacturer’s instructions. Trypan blue exclusion assay was used to assess decidual cell number and viability, which was >96%. Isolated decidual cells were suspended in 5 ml DMEM with 1% antibiotic-antimycotic and 10% FBS, and seeded onto T-25 tissue culture flasks (5×10^6^ cells/flask). After 24 h of incubation at 37°C in 5% CO_2_, attached decidual cells were washed 3X with PBS and fresh media was added. At this time, cells were all vimentin positive (mesenchymal cells), cytokeratin negative (epithelial cells) and CD45 negative (leukocytes) by flow cytometry, which assured that cultured cells were decidual cells. After an additional 24 h of incubation, supernatants were recovered, centrifuged at 2,500 rpm for 5 min, and filtered through 0.22 µm pore size membrane. One aliquot of each supernatant was tested microbiologically as mention above to assure bacteria were not present. Protein content was determined by the method of Bradford and supernatants were stored at −70°C until used. Additional supernatant sampling was taken once cultured decidual cells became confluent at the 1^st^, 2^nd^, 3^rd^, 4^th^ and 5^th^ passage (7, 14, 21, 28, 35 days of culture, respectively), and were processed as mention above.

### Cytokine and Matrix Metalloproteinase Analysis

Cytokines (IL-2, IL-4, IL-6, IL-8, IL-10, TNF-α, IL-1β) and matrix metalloproteinases (MMP-1, MMP-2, MMP-3, MMP-7, MMP-8, MMP-9) were quantified using a protein multi-array system. Supernatants were assayed for cytokines using the Bio-Plex Human Cytokine 17-plex Panel (Bio-Rad Laboratories, Hercules, CA), and for MMP’s using the Fluorokine xMAP Multiplex kits (R&D Systems, Minneapolis, MN). Supernatants were brought to 1 mg/mL using 1X PBS with 0.5% BSA and 50 µL were added to each well. A standard curve was made according to the manufacturer’s instructions. Data were acquired and analyzed on a Luminex platform using the Bio-Plex System and the Bio-Plex Manager software (Bio-Rad Laboratories, Hercules, CA).

### Prostaglandin Assay

Concentrations of PGE_2_ were measured in the supernatants using a commercially available specific enzyme immunoassay (Amersham Life Science, Buckinghamshire, UK) according to the manufacturer’s instructions.

### Statistical Analysis

Data were analyzed using the SPSS 16.0 statistics program. Inter-group comparisons were performed using an unpaired t- test (two-tailed). Intra-group comparisons were performed with one-way ANOVA with the Tukey-Kramer Multiple Comparisons Test. Statistical significance was set at the 95% level (*P*<0.05), and results were expressed as mean±SD.
